# Identification and Application of a Novel Patulin Degrading Enzyme From *Meyerozyma guilliermondii*

**DOI:** 10.1002/advs.202501146

**Published:** 2025-04-25

**Authors:** Yu Zhang, Qianhua Zhao, Solairaj Dhanasekaran, Esa Abiso Godana, Yue Zhang, Xue Bai, Qiya Yang, Hongyin Zhang

**Affiliations:** ^1^ School of Food and Biological Engineering Jiangsu University Zhenjiang 212013 P. R. China

**Keywords:** biodegradation, catalytic triad, mycotoxin, short‐chain dehydrogenase/reductase

## Abstract

Patulin (PAT), a highly toxic mycotoxin, poses significant health risks due to its contamination of fruits and their derived products. Recent biological strategies for eliminating PAT mainly focus on elucidating the molecular detoxification processes of antagonistic microorganisms using omics technologies. However, there is still a scarcity of research on the rapid screening and catalytic mechanisms of bio‐enzymes. In this study, a short‐chain dehydrogenase/reductase (MgSDR1) capable of degrading PAT is rapidly identified from *Meyerozyma guilliermondii* by integrating transcriptomics with molecular docking. MgSDR1 completely degrades PAT into *E*‐ascladiol within 2 h within the presence of reduced nicotinamide adenine dinucleotide phosphate (NADPH). Biodegradation efficiency is influenced by temperature, pH, enzyme/substrate concentrations, metal ions, and organic reagents. Notably, MgSDR1 shows efficient PAT degradation ability in fresh pear juice while maintaining key quality parameters, such as color parameters, pH, polyphenol oxidase activity, the contents of vitamin C, total phenols, titratable acidity, and soluble solids. The degradation process enhances the antioxidant capacity and enriches the aromatic compounds of the juice. Furthermore, site‐directed mutagenesis reveals the essential role of the catalytic triad (Ser174‐Tyr188‐Lys192) in MgSDR1 activity. This study provides an efficient methodology for screening PAT‐degrading enzymes and lays a theoretical foundation for the removal of mycotoxins in the food industry.

## Graphical abstract

Patulin (PAT), a hazardous mycotoxin, threatens human health. A novel yeast‐derived short‐chain dehydrogenase/reductase effectively degrades PAT into nontoxic ascladiol via catalytic triad activity. The biological detoxification process has no significant effect on the quality of fresh pear juice. The approach demonstrates significant potential for application in food safety, offering an eco‐friendly solution for mycotoxin control in the fruit processing industry.

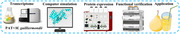

## 1 Introduction

Pear (*Pyrus* spp.), a globally significant fruit crop, is widely valued for its nutritional composition.^[^ ^1^ ^]^ However, postharvest handling, storage, and transportation render pears particularly vulnerable to *Penicillium expansum* infection, leading to blue mold disease and concomitant patulin (PAT) production, a mycotoxin.^[^ ^2^ ^]^ In addition, PAT exhibits multifaceted toxicity, including immunotoxicity, neurotoxicity, genotoxicity, and teratogenicity.^[^ ^3^ ^]^ Juice derived from contaminated pears frequently retains PAT residues, posing critical health risks to consumers.^[^ ^4^ ^]^ Consequently, PAT removal from fruit juices remains challenging owing to matrix complexity and stringent food safety requirements.

Although the physical removal of PAT is simple, the effect is inconsistent due to the interference of food matrix components.^[^ ^5^ ^]^ While chemical methods can effectively reduce PAT, they may produce harmful by‐products and damage nutritional properties.^[^ ^6^ ^]^ Therefore, biological detoxification has become a promising alternative, mainly using microorganisms or their enzymes to degrade PAT. Microbial degradation of PAT has disadvantages in food safety and operational efficiency, as it cannot avoid the risk of human infection and anaerobic growth restriction.^[^ ^7^ ^]^ Enzymatic detoxification technology has become notable for its precision, effectiveness, and eco‐friendliness, successfully degrading PAT while avoiding the introduction of secondary microbial contamination, a crucial benefit for upholding juice safety standards. These characteristics make enzymatic approaches a central area of research for the management of mycotoxins.

Microbial intracellular enzymes are acknowledged as crucial agents in PAT degradation, although their molecular processes are not yet fully understood.^[^ ^8^ ^]^ Recent studies have identified several classes of enzymes capable of degrading PAT, including lipase, aldolase, orotate phosphoribosyl transferase (OPRTase), S‐adenosylmethionine‐dependent methyltransferase, ribonucleoside diphosphate reductase, short‐chain dehydrogenase/reductase (SDR), aldo‐keto reductase and manganese peroxidase.^[^ ^9^ ^]^ For instance, lipase RL12 (100 µg mL^−1^) derived from *Ralstonia* sp. degraded 80% PAT in apple juice within 24 h at 37 °C.^[^ ^10^ ^]^ Aldolase and OPRTase derived from *Rhodotorula mucilaginosa* have demonstrated their ability to effectively degrade PAT. In apple juice, aldolase (0.7 mg mL^−1^) was able to degrade 99% of PAT (2 mg L^−1^) within 36 h, while OPRTase (0.15 g L^−1^) degraded more than 80% of PAT (1 mg L^−1^) within 18 h at 25 °C.^[^ ^11^ ^]^ Notably, CgSDR (150 µg mL^−1^) from *Candida guilliermondii* was capable of transforming PAT into *E*‐ascladiol via NADPH‐dependent catalysis, degrading 80% PAT in apple juice over 72 h.^[^ ^12^ ^]^ While juice quality parameters are maintained, these enzymes encounter practical challenges, such as extended reaction times and less than optimal efficiency, necessitating the exploration of new enzyme systems.

Computational approaches offer transformative potential for enzyme discovery and mechanistic analysis. Molecular docking allows for an accurate assessment of enzyme‐substrate interactions by calculating binding free energy and analyzing interaction patterns, while molecular dynamics simulations provide insights into conformational changes and the stability of the system.^[^ ^13^ ^]^ Based on these advantages, computer simulation technology has shown significant potential for high‐throughput screening and in elucidating catalytic mechanisms for mycotoxin‐degrading enzymes. However, related research reports are scarce, indicating a need for further investigation. Building on this foundation, the present study aims to elucidate the transcriptomic response of *M. guilliermondii* to PAT stress, identify key differentially expressed genes, and analyze PAT‐enzyme interactions through molecular docking. Furthermore, it seeks to characterize candidate enzymes for PAT degradation and develop a sustainable enzymatic system for PAT detoxification, with potential applications in enhancing food safety and juice decontamination.

The transcriptomic response mechanism of *M. guilliermondii* exposed to 10 µg mL^−1^ PAT stress was investigated using transcriptome technology. The functions of differentially expressed genes were analyzed using bioinformatics methods. Molecular docking was subsequently applied to evaluate how PAT interacts with potential degradation enzymes. Candidate enzymes were heterologously expressed in *Escherichia coli* for purification, biochemical characterization, and evaluation of their enzymatic properties, catalytic mechanisms, and potential for use in juice applications. This study provides new insights into the mechanisms of mycotoxin biodegradation. The discovery of this novel degrading enzyme lays a foundation for constructing an immobilized enzyme system capable of achieving self‐sustaining cofactor recycling, thereby achieving the efficient removal of mycotoxin in the food industry.

## 2 Result

### 2.1 Transcriptome Sequencing Analysis

**Figure 1:**
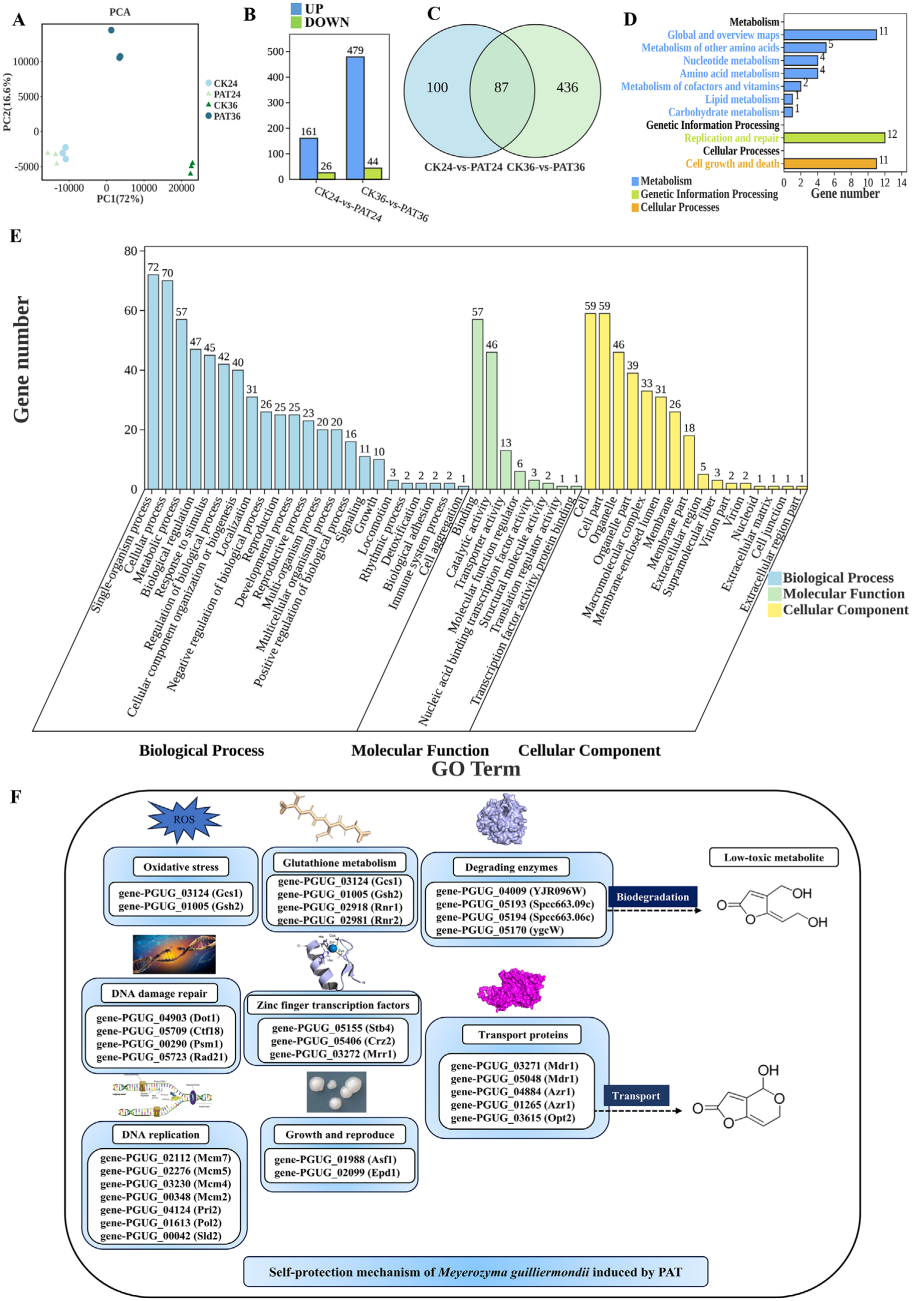
A) GO enrichment analysis of 87 differentially expressed genes. B) KEGG enrichment analysis of 87 differentially expressed genes. C) Self‐protection mechanism of *Meyerozyma guilliermondii* induced by PAT.

In total, twelve libraries were built. **Figure** **1**A presents the principal component analysis (PCA) of these libraries, illustrating that samples from the same group cluster together with similar PC1 values, which explain the majority of the variation (72%). As shown in Figure 1B, compared to the control group, the treatment group exhibited significant up‐regulation of 161 genes and down‐regulation of 26 genes at 24 h. At 36 h, 479 genes were significantly up‐regulated, while 44 genes were significantly down‐regulated in the treatment group. There were 87 common genes between CK24 versus PAT24 and CK36 versus PAT36 (Figure 1C). Kyoto Encyclopedia of Genes and Genomes (KEGG) enrichment analysis of these 87 differentially expressed genes (DEGs) showed that they were enriched in three categories: metabolism (7 subcategories), genetic information processing (1 subcategory), and cellular process (1 subcategory). Amino acid metabolism, carbohydrate metabolism, other amino acid metabolism, replication, and repair might be related to the growth, stress response regulation, and stress resistance of *M. guilliermondii* (Figure 1D). Gene Ontology (GO) enrichment analysis of these 87 DEGs revealed that they were enriched in three primary categories: biological processes (23 subcategories), molecular functions (8 subcategories), and cellular components (16 subcategories). The cell membrane components and cell membrane subcategories might be involved in PAT transport by *M. guilliermondii*. The transport activity might be related to the transport function of the membrane. The catalytic and oxidative activities may be involved in the stress resistance of *M. guilliermondii*. The biological regulation, signaling, detoxification and response to stimulus subcategories might be involved in PAT removal by *M. guilliermondii* (Figure 1E).

According to the annotation information of the GO and KEGG databases, the DEGs were further classified, and it was identified that 29 genes were potentially linked to the self‐protection mechanism of *M. guilliermondii* induced by PAT. These genes are mainly associated with oxidative stress, Glutathione (GSH) metabolism, DNA damage repair, DNA replication, growth and reproduction, resistance and drug resistance, degrading enzymes, and zinc finger transcription factors. The relevant data for these genes are shown in Figure 1F and Table S1 (Supporting Information).

### 2.2 Molecular Docking of Proteins and PAT

The accuracy of the protein structural model was evaluated using ERRAT and PROCHECK software.^[^ ^14^ ^]^ ERRAT identified incorrect non‐bonded atom contacts with a quality score exceeding 90%, confirming the suitability of the models. Analysis of the Ramachandran plot revealed that ≈90% of the residues were in the most favored regions, indicating proper Psi/Phi dihedral angles in the predicted 3D structure. This finding supported the applicability of these models for further molecular docking analysis (Figure S1, Supporting Information).

**Figure 2:**
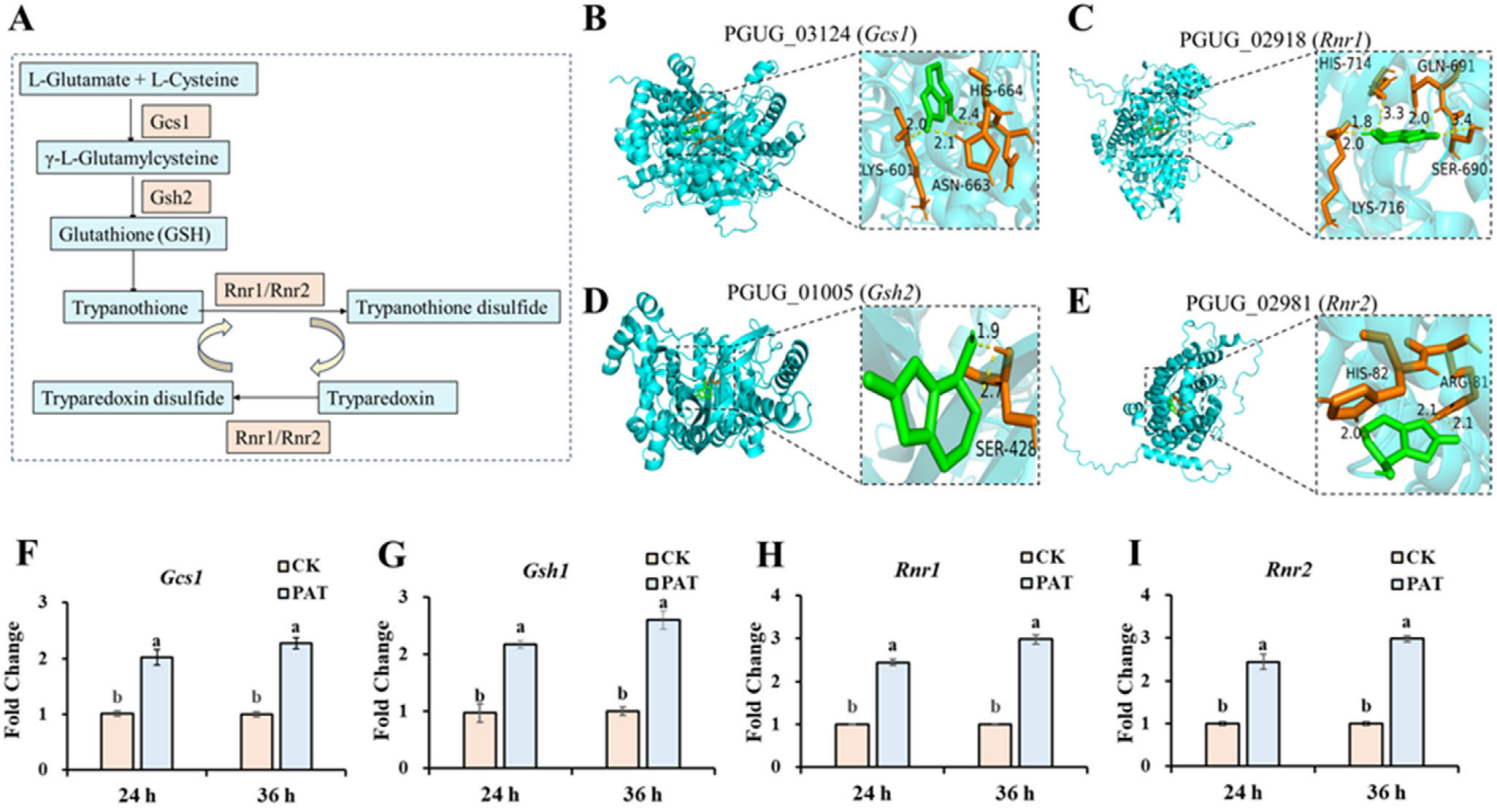
Molecular characterization of glutathione metabolic pathway‐related genes. A) differentially expressed genes participated in the glutathione metabolic pathway. B–E) Molecular docking of glutathione metabolic pathway‐related enzymes with PAT. F‐I) The determination of the expression of key genes related to glutathione metabolic pathway by RT‐qPCR. Means with different letters show significant differences at *p* < 0.05 according to the independent sample *t*‐test.

In the GSH metabolic pathway, four genes were significantly up‐regulated, including *Gcs1* (encoding glutamate‐cysteine ligase catalytic subunit), *Gsh2* (encoding GSH synthase), *Rnr1* and *Rnr2* (encoding ribonucleoside‐diphosphate reductase subunit M1 and M2) (**Figure** **2**A). The binding of the above four proteins to PAT was simulated by molecular docking. As shown in Figure 2B–E, enzymes associated with the GSH metabolic pathway can form hydrogen bonds with PAT, with these bonds ranging from 1.8 to 3.4 Å in length. Moreover, RT‐qPCR analysis revealed that the expression levels of these four genes were significantly elevated. (Figure 2F–I).

**Figure 3:**
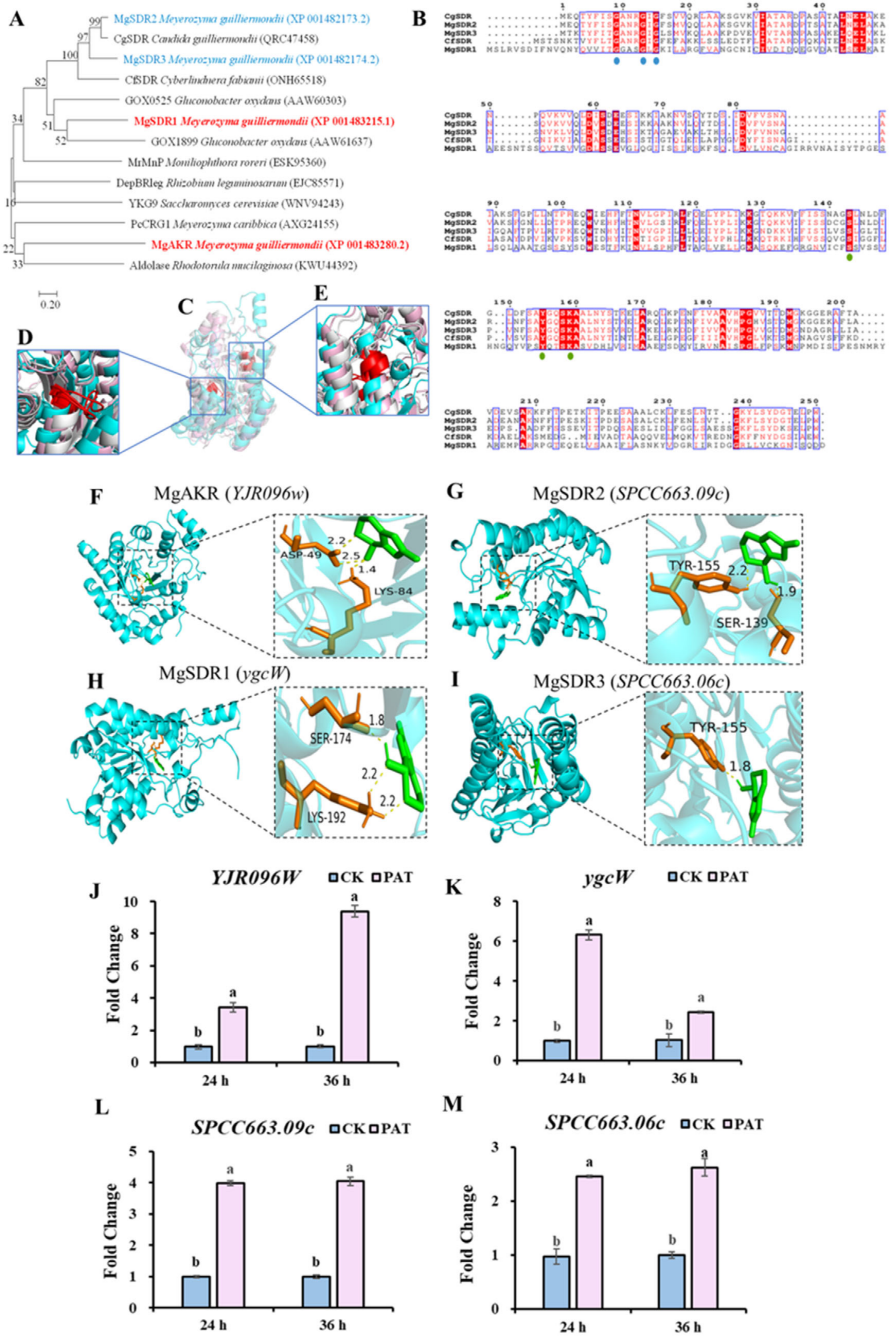
Molecular characterization of potential degrading enzymes. A) Phylogenetic tree of MgSDR1 and known PAT‐degrading enzymes. The GenBank accession number is listed in brackets. B) Sequence alignment of different SDRs. Extreme conserved residues of active sites are shown in the green circle, and extreme conserved residues of cofactor‐binding sites are shown in the blue circle. C) The 3D structure comparison of MgSDR1 and other SDRs. The blue model represents MgSDR1, the pink model represents MgSDR2, and the white model represents MgSDR3. D) The local conformation of the cofactor binding region. E) The local conformation of the active sites of MgSDR1. F–I) Molecular docking of potential degrading enzymes with PAT. J–M) The determination of the expression of key genes related to potential degrading enzymes by RT‐qPCR. Means with different letters show significant differences at *p* < 0.05 according to the independent sample t‐test.

Molecular docking was used to screen proteins with catalytic and redox activities capable of binding to PAT from the DEGs. Combined with the related literature, a total of four potential degradation enzymes were screened, and their coding genes were significantly up‐regulated. In the potential degrading enzymes, four genes were significantly up‐regulated; *YJR096* *W* encodes aldo‐keto reductase (MgAKR), and *ygcW*, *SPCC663.09c*, and *SPCC663.06c* encode MgSDR1, MgSDR2 and MgSDR3, respectively. The phylogenetic tree analysis of the four degrading enzymes and the degrading enzymes reported in the literature revealed that MgAKR and MgSDR1 exhibited low similarity to the previously reported degrading enzymes, suggesting their potential novelty (**Figure** **3**A). The function of MgAKR has been reported in previous studies. Consequently, our primary focus was on investigating the function of MgSDR1. MgSDR1 can be aligned to the cofactor binding site (GXXXGXG) and active site (YXXXK) of the SDRs family (Figure 3B–E). As shown in Figure 3F–I, potential degrading enzymes can bind to PAT through hydrogen bonds, with these bonds ranging from 1.8 to 2.5 Å in length. Moreover, RT‐qPCR analysis revealed that the expression levels of these four genes were significantly elevated (Figure 3J–M).

### 2.3 Protein Expression

The expression of these proteins is shown in Figure S2 (Supporting Information). The molecular weight of MgAKR, MgSDR1, MgSDR2 and MgSDR3 with the N‐terminal 6 × His‐Tag was ≈38, 40, 36, and 35 kDa, respectively. The physical and chemical properties of potential degrading enzymes are shown in Table S2 (Supporting Information). The prokaryotic expression results indicated that MgSDR1 and MgAKR were expressed in a soluble form, whereas MgSDR2 and MgSDR3 were predominantly found in inclusion bodies. The degradation effect of *E. coli* crude enzyme solution expressing MgSDR1 on PAT was better than that of the control group without inducing protein expression (Figure S3, Supporting Information).

### 2.4 Molecular Dynamics Simulation

**Figure 4:**
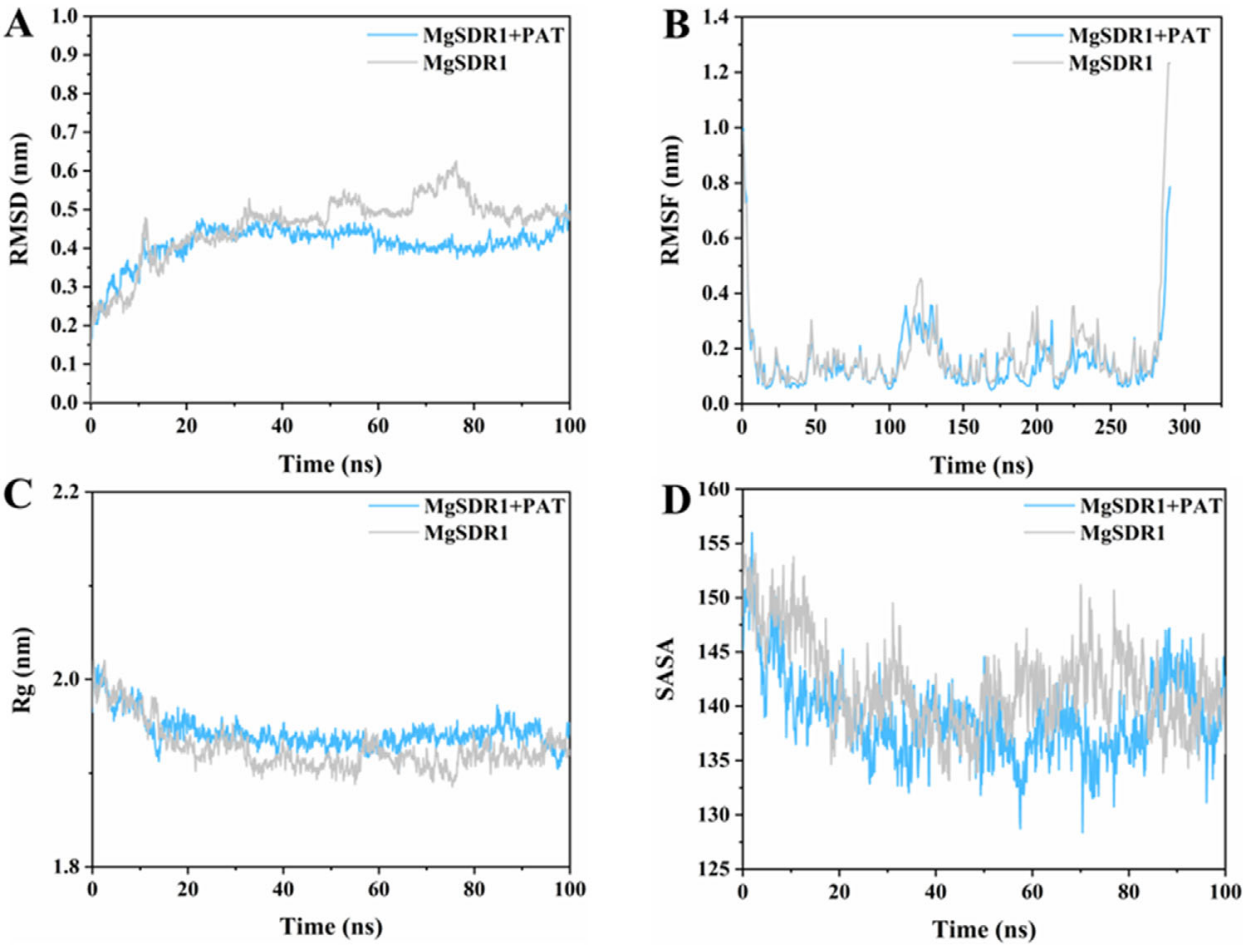
Molecular dynamics simulation of the MgSDR1‐PAT complex. A) Root mean square deviation (RMSD). B) Root mean square fluctuation (RMSF). C) Radius of gyration (Rg). D) Solvent accessible surface area (SASA).

The conformational change and stability of the MgSDR1‐PAT complex were analyzed by molecular dynamics simulations. The root mean square deviation (RMSD) analysis indicated that the MgSDR1‐PAT complex experienced minor fluctuations during the first 10 ns, mainly attributed to PAT binding, before achieving stability for the rest of the simulation. The MgSDR1‐PAT complex exhibited a lower RMSD compared with MgSDR1 alone, suggesting a strong association between MgSDR1 and PAT (**Figure** **4**A). Overall, the root mean square fluctuation (RMSF) of the MgSDR1‐PAT complex was lower compared to that of MgSDR1 during the 100 ns simulation period, suggesting that the MgSDR1‐PAT complex exhibited greater stability (Figure 4B). Throughout the simulation, slight variations in solvent‐accessible surface area (SASA) were observed between the MgSDR1‐PAT complex and MgSDR1. This suggests that PAT binding may influence the surface accessibility of MgSDR1 to some extent, although the overall impact was minimal (Figure 4C). Figure 4D illustrates that the radius of gyration (Rg) of the MgSDR1‐PAT complex was slightly altered compared to MgSDR1. However, the MgSDR1‐PAT complex exhibited reduced Rg fluctuations overall, suggesting that while PAT binding had minimal impact on the overall compactness of MgSDR1, it could contribute to maintaining the stability of MgSDR1.

### 2.5 Effect of Concentration of NADPH on PAT Degradation by MgSDR1 and Verification of the Degradation Product

**Figure 5:**
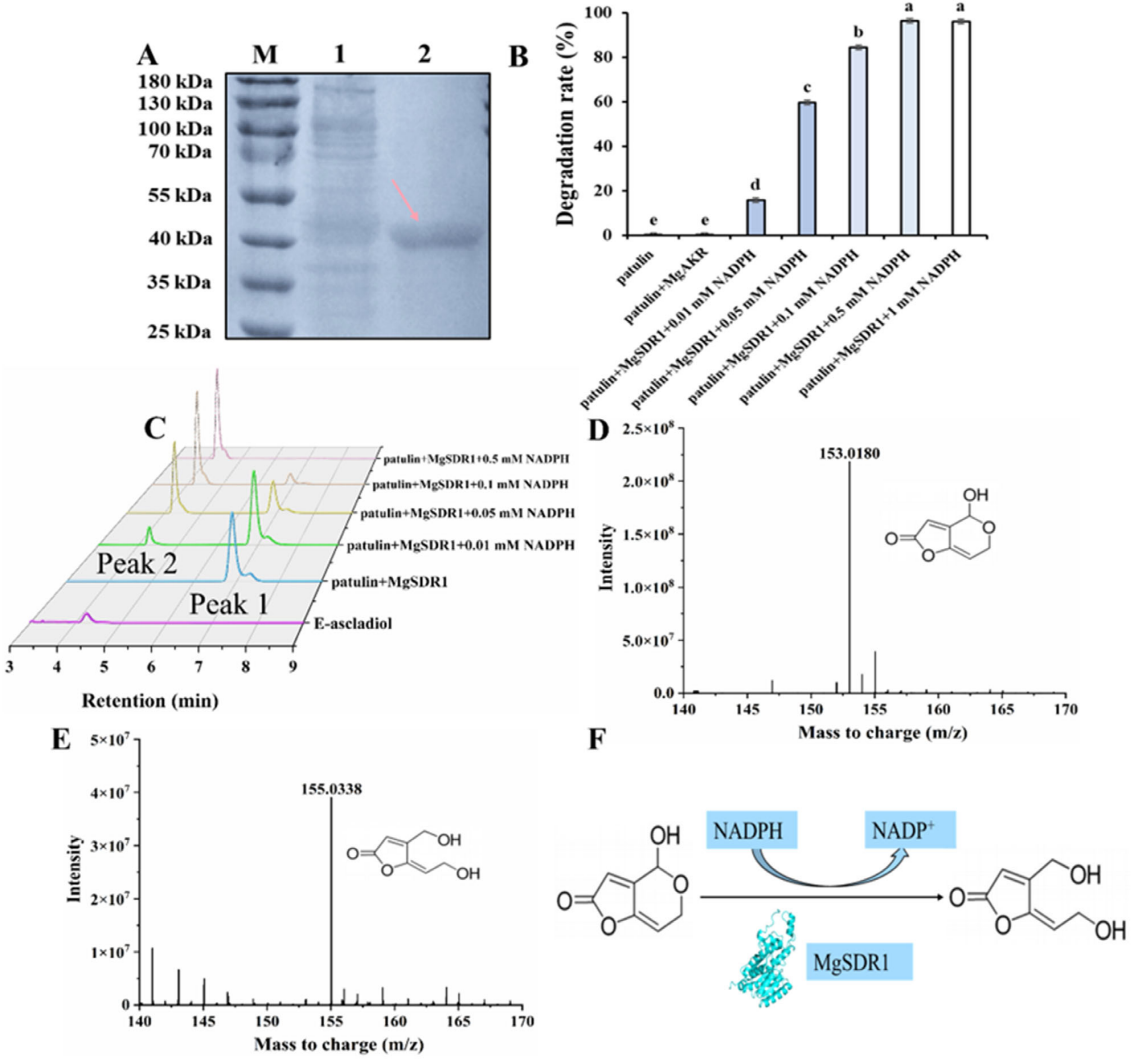
Effect of NADPH concentration on the degradation of PAT by MgSDR1 and verification of the PAT degradation product. A) SDS‐PAGE analysis of MgSDR1. Lane 1: PageRuler prestained molecular weight marker, Lane 2: crude extract, Lane 3: fraction eluted with 500 mM imidazole. The arrow indicates the position of the recombinant MgSDR1. B) The effect of NADPH concentration on the degradation efficiency of PAT by MgSDR1. C) HPLC analysis of degradation samples. D‐E) LC‐ESI/MS analysis of PAT and its product. F) Biological transformation of PAT by MgSDR1. The data were expressed as means ± SD. Means with different letters show significant differences at *p* < 0.05 according to Duncan's multiple range test.

MgSDR1 underwent additional purification to achieve a single distinct target band (**Figure** **5**A). NADPH is a cofactor of many enzymatic reactions. When its concentration reached 0.5 mM, PAT was undetectable at 2 h (Figure 5B). In addition, we analyzed the products of PAT degradation by MgSDR1. High‐performance liquid chromatography (HPLC) showed that PAT peaked at 6.9 min (peak 1), and a new product peak (peak 2) was generated at 4.2 min (Figure 5C). The molecular weights of PAT and its degradation product were identified by liquid chromatography‐electrospray Ionization‐mass spectrometry (LC‐ESI/MS). In the negative ion mode, the molecular weight of PAT and its degradation product was 153.0180 and 155.0338, respectively. Overall, the conversion of PAT to *E*‐ascladiol by MgSDR1 exhibited strict reduced nicotinamide adenine dinucleotide phosphate (NADPH) dependence (Figure 5D–F).

### 2.6 Effect of Reaction Conditions, Metal Ions and Organic Reagents on PAT Degradation by MgSDR1

**Figure 6:**
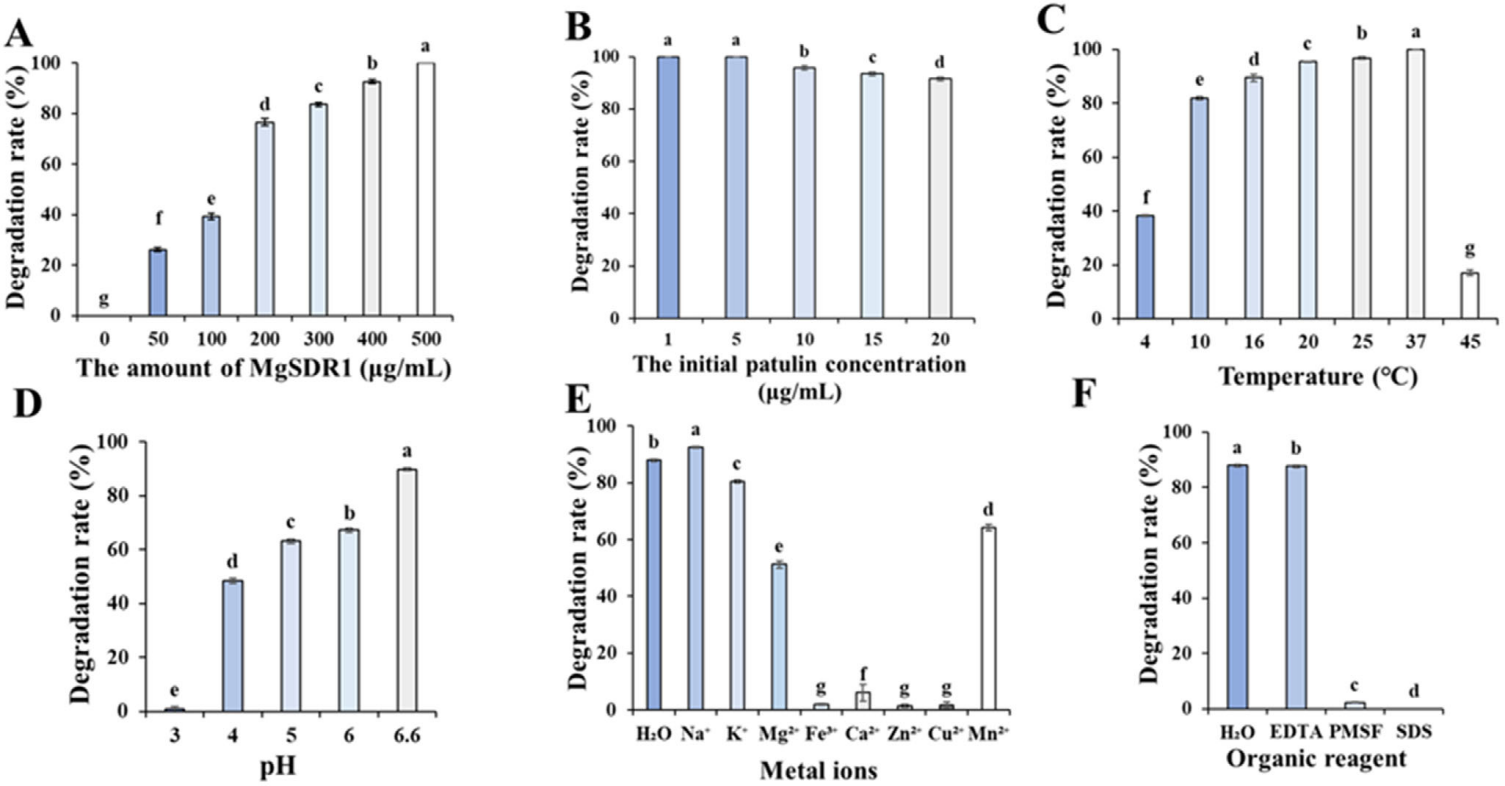
The effect of reaction conditions on the degradation of PAT by MgSDR1. A) The amount of MgSDR1. B) The initial PAT concentration. C) Temperature. D) pH. E) Metal ions. F) Organic reagents. The data were expressed as means ± SD. Means with different letters show significant differences at *p* < 0.05 according to Duncan's multiple range test.

The biodegradation process was easily affected by temperature, pH, MgSDR1 concentration, and initial PAT concentration. **Figure** **6**A showed that the MgSDR1 concentration was positively correlated with the degradation rate of PAT. When MgSDR1 concentration was 500 µg mL^−1^, the degradation rate of PAT was 100%. Figure 6B showed that when the initial concentration of PAT was 1–5 µg mL^−1^, it was completely degraded within the same time frame. However, when the PAT initial concentration was 20 µg mL^−1^, the degradation rate was 91.44% within 2 h. Figure 6C showed that MgSDR1 exhibited the best catalytic activity between 25 and 37 °C, achieving a complete degradation rate of 100%. Figure 6D showed that pH significantly affected the degradation rate of PAT by MgSDR1. Specifically, the degradation rate of PAT by MgSDR1 was below 1% at pH 3.0, reached 48% at pH 4.0, and achieved 90% at pH 6.6.

Metal ions and organic compounds could significantly affect the activity of the enzyme. Figure 6E showed that the presence of Fe^3+^, Cu^2+^, Ca^2+^, and Zn^2+^ has a strong inhibitory effect on the activity of MgSDR1, reducing its activity to less than 10%. The enzyme activity remained above 50% in the presence of K^+^, Mg^2+^, and Mn^2+^. In contrast, Na^+^ could promote the degradation of PAT by MgSDR1. Figure 6F showed that PMSF and SDS significantly inhibited MgSDR1‐mediated PAT degradation, while EDTA had little effect on the activity of MgSDR1.

### 2.7 PAT Degradation Assay in Fresh Pear Juice and Quality Determination

**Figure 7:**
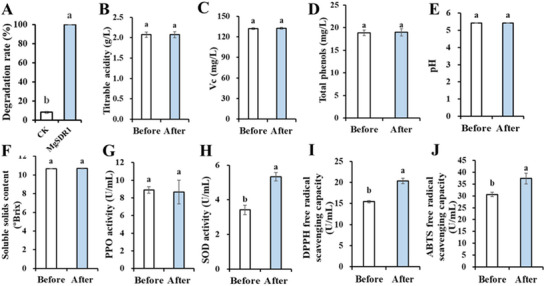
A) Degradation of PAT in fresh pear juice by MgSDR1. B–J) Quality parameters of fresh pear juice. The data were expressed as means ± SD. Means with different letters show significant differences at *p* < 0.05 according to the independent sample *t*‐test.

The degradation of PAT by MgSDR1 was verified in the fresh pear juice. PAT (10 µg mL^−1^) in fresh pear juice was completely degraded within 12 h, with a degradation rate of 100% (**Figure** **7**A). In view of the higher degradation efficiency of PAT in fresh pear juice by MgSDR1 and its considerable application prospect, we measured the quality parameters of fresh pear juice. The biodegradation process had no significant effect on pH, polyphenol oxidase (PPO) activity, the contents of titratable acidity, vitamin C (Vc), soluble solids, and total phenol (Figure 7B–G). After biodegradation, superoxide dismutase (SOD) activity, 2,2‐diphenyl‐1‐picrylhydrazyl (DPPH) and 2,2′‐azino‐bis‐(3‐ethylbenzothiazoline‐6‐sulfonic acid (ABTS) free radical scavenging capacity of juice were significantly improved. The above results showed that the biodegradation process could improve the antioxidant capacity (Figure 7H–J). The fresh pear juice exhibited a yellowish‐brown color. The color parameters (L*, a*, b*), total color difference (ΔE*), cloudiness, and browning index of the samples remained consistent before and after undergoing biological degradation. This indicates that the PAT degradation process did not alter the color, cloudiness, or degree of browning in the fresh pear juice (Table S3, Supporting Information).

The changes in aroma compounds before and after biodegradation were determined by gas chromatography/mass spectrometer (GC/MS). According to the GC/MS results, there was an increase in the levels of hexanal, 1‐hexanol, 3‐Hexen‐1‐ol, acetate, (*Z*)‐, acetic acid, hexyl ester, and decanal, while nonanal and 1‐dodecanol showed a slight decrease. Overall, the biodegradation process promoted the formation of esters, aldehydes, and alcohols (Table S4, Supporting Information).

### 2.8 Site‐Directed Mutation

**Figure 8:**
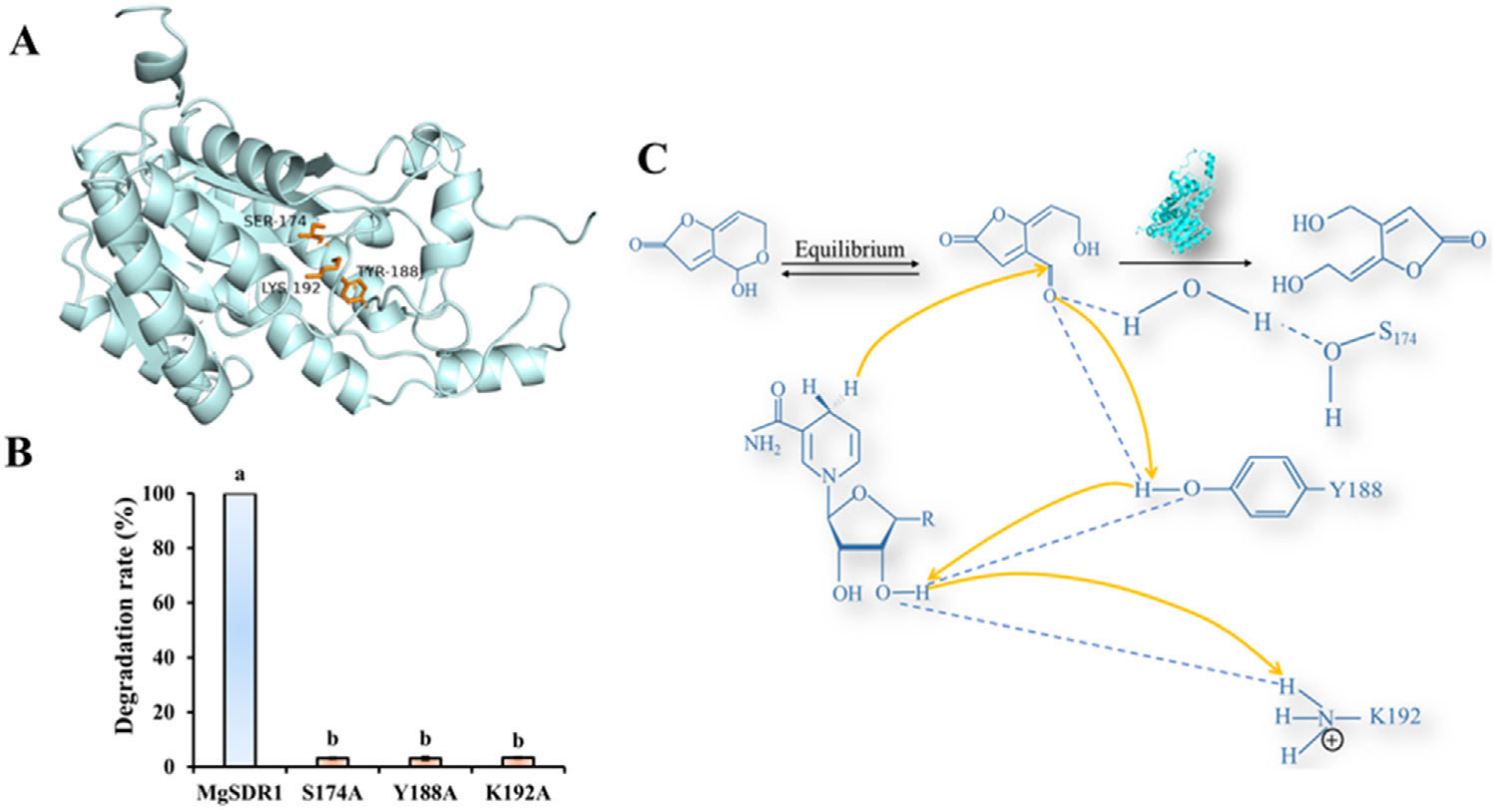
Site‐directed mutation of MgSDR1. A) Catalytic triad Ser‐Tyr‐Lys of MgSDR1, MgSDR2 and MgSDR3. The blue stick belongs to MgSDR1, and the orange stick belongs to MgSDR2 and MgSDR3. B) Effect of MgSDR1^S174A^, MgSDR1^Y188A^, and MgSDR1^K192A^ on the degradation of PAT. C) Catalytic mechanism of MgSDR1 in the detoxification process of PAT. The data were expressed as means ± SD. Means with different letters show significant differences at *p* < 0.05 according to Duncan's multiple range test.

MgSDR1, MgSDR2, and MgSDR3 possess a conserved catalytic triad (**Figure** **8**A). The site‐directed mutagenesis was performed to investigate the role of the key catalytic triad in PAT degradation by MgSDR1. The active sites of MgSDR1 (Ser174, Tyr188, and Lys192) were replaced with Ala. As a result, the mutated proteins (MgSDR1^S174A^, MgSDR1^Y188A^ and MgSDR1^K192A^) exhibited a significantly decreased PAT degradation rate, with near‐complete loss of activity (Figure 8B). Based on these findings, we propose a mechanism for PAT degradation. The Tyr side chain acts as a catalytic base, and the pKa of the catalytic base hydroxyl group can be reduced by interacting with the ribose hydroxyl group of NADPH, positively charged lysine and catalytic serine. In addition, the side chain of the catalytic Ser may interact with the substrate PAT, thereby stabilizing it in a reasonable conformation within the binding pocket of the base strategy. The catalytic process starts with the transfer of protons from the hydroxyl group of Tyr to the carbonyl oxygen atom of PAT. Subsequently, the hydrogen atom of NADPH is transferred to the carbonyl carbon, resulting in the conversion of the carbonyl group of PAT to a hydroxyl group (Figure 8C).

## 3 Discussion

Mycotoxins represent critical toxic metabolites posing substantial risks to food and feed safety.^[^ ^15^ ^]^ Microbial detoxification strategies leverage natural or engineered bacterial metabolism to neutralize these toxins, circumventing energy‐intensive equipment or chemical reagents while aligning with green chemistry principles and minimizing secondary contamination risks. Investigating microbial degradation mechanisms is thus imperative for advancing biotechnological solutions. In this study, GO enrichment analysis demonstrated that PAT exposure upregulated catalytic and transport activity‐related proteins in *M. guilliermondii*, facilitating detoxification. Mechanistic studies revealed PAT toxicity counteraction through enhanced expression of genes involved in oxidative stress response, GSH metabolism, DNA repair, antimicrobial resistance, and detoxification enzymes.^[^ ^13^, ^16^ ^]^ Notably, four DEGs were significantly enriched in the glutathione metabolism pathway (KEGG map00480). Key enzymes included Gcs1 (catalyzing γ‐L‐glutamylcysteine synthesis) and Gsh2 (driving GSH production), alongside Rnr1/Rnr2 regulating redox homeostasis via trypanothione interconversion.^[^ ^13a^ ^]^ GSH, a tripeptide composed of glutamic acid, cysteine, and glycine, serves as a vital antioxidant that scavenges free radicals and mitigates oxidative stress.^[^ ^17^ ^]^ Furthermore, GSH participates in biotransformation by conjugating with electrophilic toxins such as PAT, facilitating their neutralization and excretion.^[^ ^18^ ^]^ Supporting this, studies on *Saccharomyces cerevisiae* demonstrated that GSH reacts with PAT in cellular lysates, generating four covalent adducts (dihydropyranone‐GSH‐PAT, c‐GSH‐PAT, l‐GSH‐PAT, and l, c‐GSH2‐PAT), as identified by LC‐MS/MS.^[^ ^19^ ^]^ These findings establish GSH metabolism as a central detoxification axis in *M. guilliermondii*.

The identification of potential PAT‐degrading enzymes was achieved through transcriptome analysis coupled with molecular docking strategies.^[^ ^13a^ ^]^ In this study, four candidate enzymes were screened using these integrated approaches, with MgAKR and MgSDR1 demonstrating significant evolutionary divergence from previously reported homologs. Aldo‐keto reductases (AKRs), key members of the oxidoreductase superfamily, utilize NADPH as a cofactor to catalyze the reduction of ketones and aldehydes to alcohols, playing essential roles in osmotic regulation, reactive aldehyde detoxification, and secondary metabolism.^[^ ^20^ ^]^ Notably, MgAKR from *M. guilliermondii* exhibited exceptional PAT degradation capacity, achieving complete decomposition of 15 µg mL^−1^ PAT within 4 h under 0.5 mm NADPH.^[^ ^21^ ^]^ MgSDR1 belongs to the NADPH‐dependent oxidoreductase family characterized by conserved sequence motifs and catalytic mechanisms. Despite sharing lower than 20% sequence identity with known PAT‐degrading enzymes, MgSDR1 retained structural homology with SDR family members, particularly in the cofactor‐binding and active‐site domains. Multiple sequence alignment revealed the presence of the N‐terminal glycine‐rich GXXXGXG motif, a hallmark of classical SDRs critical for coenzyme binding.^[^ ^22^ ^]^

Molecular dynamics simulation serves as a pivotal technique for studying the stability and kinetic characteristics of proteins in aqueous solutions. It can observe the dynamic process of protein‐substrate binding at the atomic level in detail, including the steps of binding site recognition, substrate proximity, binding, and release. In this study, the four indicators (RMSD, RMSF, SASA, and Rg) indicate that MgSDR1 could be closely combined with PAT. Our future efforts will be directed toward identifying additional degrading enzymes, creating a database through molecular dynamics simulation and molecular docking, and assessing their degradation capabilities.

Recent studies have highlighted several SDRs capable of degrading PAT, including CgSDR from *Candida guilliermondii*, GOX0525/GOX1899 from *Gluconobacter oxydans*, and BsSDRs from *Bacillus subtilis*. MgSDR1 catalyzes the NADPH‐dependent conversion of PAT to *E*‐ascladiol. The degradation efficiency is influenced by parameters such as enzyme dosage, substrate concentration, temperature, and pH.^[^ ^23^ ^]^ MgSDR1 exhibited peak activity at 37 °C, which is consistent with the optimal temperature range of CgSDR (37–45 °C) and BsSDRs (35–40 °C). Notably, MgSDR1 retained more than 90% degradation activity at pH 6.6 and 50% at pH 4.0, which is crucial for the application of acidic juice. In contrast, BsSDRs show only 20% degradation activity at pH 4.0, limiting their utility.^[^ ^24^ ^]^ Furthermore, MgSDR1 could completely degrade PAT in the buffer system within 2 h, outperforming other SDRs. These properties position MgSDR1 as a prime candidate for industrial PAT detoxification. In the future, the degradation enzyme can be further modified to improve its pH adaptability. For example, ancestral sequence reconstruction has identified variable regions where amino acid substitutions may optimize pH tolerance without functional loss.^[^ ^25^ ^]^

The toxicity of PAT is primarily attributed to its hemiacetal and lactone ring structures, and the destruction of these structures will reduce its toxicity.^[^ ^7^ ^]^ PAT degradation yields four major products: *E*‐ascladiol, *Z*‐ascladiol, desoxypatulinic acid (DPA), and hydroascladiol. The hemiacetal structure is destroyed during the biodegradation of PAT into *E*‐ascladiol, *Z*‐ascladiol and DPA, while the lactone ring is disrupted during biodegradation to hydroascladiol. *E*‐ascladiol and *Z*‐ascladiol naturally interconvert through non‐enzymatic mechanisms.^[^ ^26^ ^]^ Microorganisms such as *S. cerevisiae*, *G. oxydans*, *Pichia guilliermondii*, and *Kodamaea ohmeri* facilitate PAT transformation into *E*/*Z*‐ascladiol.^[^ ^27^ ^]^ Studies indicate that ascladiol is non‐toxic to human liver, kidney, intestine, and immune system cells.^[^ ^28^ ^]^ Additionally, while PAT exerts toxic effects on the porcine intestinal mucosa, its degradation product ascladiol shows no toxicity.^[^ ^29^ ^]^ Current evidence thus identifies ascladiol as a non‐toxic PAT degradation product. However, comprehensive long‐term toxicological studies are imperative to assess the safety of enzymatic byproducts and ensure food quality preservation.

Assessing the impact of biological detoxification on fruit juice quality is critical for practical application. Color serves as a key indicator of microbial safety and sensory attributes during juice processing, storage, and consumption.^[^ ^30^ ^]^ The MgSDR1‐mediated PAT biodegradation preserved essential quality parameters of fresh pear juice, without negatively impacting its color, cloudiness, or browning degree. Furthermore, pH, soluble solids, titratable acidity, total phenols, and Vc levels remained statistically unchanged. PPO, a pivotal enzyme in enzymatic browning, catalyzes the oxidation of phenolic compounds, leading to undesirable black/brown pigmentation.^[^ ^31^ ^]^ There was no significant change in PPO activity before and after biodegradation, confirming that the detoxification process did not accelerate browning. Notably, the detoxification process significantly enhanced the antioxidant capacity of the juice, which was manifested by the increase in SOD activity and DPPH/ABTS free radical scavenging rate.^[^ ^32^, ^33^ ^]^ In addition, the effect of the biodegradation process on the aroma of juice should also be considered. Hexanal imparts a grassy and green leaf aroma, and a significant increase in content may enhance the freshness of the juice. 1‐Hexanol has a fruity and herbaceous flavor, which may increase the complexity of the fruity flavor.^[^ ^34^ ^]^ The compound 3‐Hexen‐1‐ol, acetate, (*Z*)‐ has the sweet flavor of the fruit, and the increase of their content may strengthen the typical fruit flavor characteristics of fruit juice.^[^ ^35^ ^]^ Acetic acid, hexyl ester is the key compound of aroma characteristics of pear juice.^[^ ^36^, ^37^ ^]^ Decanal has floral and citrus aromas, increasing its level, which may give the juice a brighter aroma. 1‐Dodecanol usually has a lighter smell, and the reduction has little effect on the overall aroma. A slight decrease in nonanal may weaken the floral and citrus aromas, but the overall effect remains minimal.^[^ ^38^ ^]^

Members of the NADPH‐dependent SDR superfamily exhibit a conserved catalytic mechanism, with the classical catalytic triad (Ser‐Tyr‐Lys) playing a pivotal role in catalysis.^[^ ^39^ ^]^ Site‐directed mutagenesis of the MgSDR1 catalytic triad (Ser174, Tyr188, and Lys192) to Ala residues resulted in more than 95% loss of enzymatic activity, underscoring its functional necessity. Kinetic characterization revealed a Michaelis constant (*K*m) of 1.42 mm and a catalytic efficiency (*k*cat/*K*m) of 8.66 s^−1^ M^−1^ (Table S5, Supporting Information), highlighting the substrate affinity and turnover capacity of MgSDR1. Hemiacetal and acetaldehyde are in equilibrium in solution. The latter contains carbonyl fragments, which are more easily protonated.^[^ ^40^ ^]^ The detoxification process catalyzed by MgSDR1 may be derived from the aldehyde form of PAT. The tertiary catalytic site formed by residues (Ser174, Tyr188, and Lys192) is the key to realizing the function of MgSDR1, and its catalytic process follows a conservative electron transfer and reaction mechanism.^[^ ^24^, ^40^, ^41^ ^]^ Future studies will prioritize resolving the crystal structure of MgSDR1 to elucidate conformational dynamics, substrate recognition patterns, and precise spatial arrangements of the catalytic triad. These structural insights will validate the proposed mechanism and inform engineering strategies to enhance catalytic performance.

The biocatalytic enzyme MgSDR1 demonstrates exceptionally high efficiency in PAT degradation, providing an environmentally sustainable alternative to traditional chemical detoxification methods. Its rapid catalytic kinetics and strict substrate specificity enable precise mitigation of PAT under optimized conditions, which aligns with green chemistry principles and circular economy objectives. However, industrial deployment faces critical challenges: 1) Structural instability under extreme industrial conditions (elevated temperatures, pH extremes, high salinity) requiring stability enhancements through protein engineering or immobilization strategies; 2) High production costs associated with extraction/purification workflows, potentially addressable through genetic engineering for enhanced expression yields and operational stability; 3) Restricted substrate specificity that limits utility in multi‐mycotoxin systems, necessitating the development of synergistic enzyme consortia or engineered broad‐spectrum variants; 4) Inefficient post‐reaction enzyme recovery, solvable through magnetic‐nanocarrier immobilization strategies to enable reuse and streamline bioprocessing. Although MgSDR1 exemplifies a transformative approach to PAT degradation, overcoming its industrial limitations requires interdisciplinary integration of enzymology, materials science, and synthetic biology.

## 4 Conclusion

In this study, transcriptome analysis revealed significant upregulation of genes involved in oxidative stress, GSH metabolism, transporters, DNA damage repair, DNA replication, growth and reproduction, and zinc finger transcription factors to counteract PAT stress in *M. guilliermondii*. Moreover, MgSDR1, a novel PAT‐degrading enzyme, could efficiently convert PAT into *E*‐ascladiol using NADPH as a cofactor. The optimal temperature range was 25–37 °C, and within an acidic pH range, the PAT degradation rate by MgSDR1 increased with rising pH levels. Notably, the classical catalytic triads (Ser174‐Tyr188‐Lys192) played an indispensable role during the catalytic process. More importantly, during biological detoxification, MgSDR1 demonstrated effective PAT removal capacity in fresh pear juice without compromising its quality. PAT remains stable in acidic conditions and is highly water‐soluble, facilitating its distribution during water‐based food processing. Therefore, MgSDR1 offers a promising solution for the efficient removal of PAT. Additionally, this study provides a method for the rapid identification of PAT‐degrading enzymes, which lays an important theoretical foundation for research on microbial mycotoxin removal mechanisms.

## 5 Experimental Section

### Reagents

The commercial PAT and *E*‐ascladiol standards were purchased from PriboLab (Qingdao, China). HPLC‐grade methanol and acetonitrile were purchased from TEDIA (Cincinnati, Ohio, USA).

### Culture of M. Guilliermondii and Preparation of Samples

The strain *M. guilliermondii*, which was identified, isolated, and purified from the surface of decaying pears, is preserved at the China Center for Type Culture Collection, with the preservation number M2017270. First, the glycerol stock of the strain stored in an ultra‐low‐temperature freezer was inoculated into Nutrient yeast dextrose broth (NYDB) medium (8 g L^−1^ nutrient broth, 5 g L^−1^ yeast extract, and 10 g L^−1^ glucose) at a 2% volume ratio overnight. Second, a sterile loop was employed to spread an adequate quantity of the suspension onto a Nutrient yeast dextrose agar (NYDA) plate (NYDB supplemented with 2% agar), which was then incubated at 28 °C for 2–3 d. Finally, a well‐developed colony was selected, inoculated into NYDB medium, and cultured at 28 °C with agitation at 180 rpm for 20 h. The culture was centrifuged to remove the medium, washed three times with sterile water, and adjusted to 1 × 10^8^ cells per mL. Finally, 1 mL of yeast culture was inoculated into 50 mL of NYDB medium with one setup serving as the control (CK) without PAT and the other as the experimental group (PAT). After incubation at 28 °C, 180 rpm for 24 and 36 h, the medium was removed by centrifugation at 4 °C, 8000 × *g* for 10 min. The cells were then rinsed three times with sterile water, frozen in liquid nitrogen, and promptly stored at −80 °C.

### Transcriptome Sequencing

The samples frozen at −80 °C in Section 2.2 were sent to Gene Denovo Biotechnology Co. Ltd. (Guangzhou, China) for high‐throughput sequencing. Omicsmart (http://www.omicsmart.com) was used to perform bioinformatic analysis. Raw reads were analyzed with Fastp to eliminate low‐quality sequences, yielding clean reads that were mapped onto the reference genome (GCF_000 149425.1). DEGs were identified with |log_2_Fold Change| ≥ 1 and FDR < 0.05. GO and KEGG were used to analyze the functions of the identified DEGs.

### RT‐qPCR Analysis of Related DEGs

Specific genes related to glutathione (GSH) metabolism and degrading enzymes were selected for RT‐qPCR. Primers were designed by the Sangon Biotech website (https://store.sangon.com/newPrimerDesign) and listed in Table S6 (Supporting Information). Total RNA was extracted from the samples using a Yeast Total RNA Isolation Kit (Sangon Biotech (Shanghai) Co. Ltd., China). Then, the RNA was reverse transcribed into cDNA using HiFiScript gDNA Removal RT MasterMix (Vazyme, Nanjing, China). RT‐qPCR was performed using the three‐step method with 2 × ChamQ Blue Universal SYBR qPCR Master Mix (Vazyme, Nanjing, China) on a LightCycler96 instrument (Roche Diagnostics GmbH, Germany). The relative expression level was determined by the 2^−ΔΔCt^ following a modified version of the protocol by Livak et al.^[^ ^42^ ^]^

### Molecular Docking of Proteins and PAT

Multiple sequence alignments were carried out using CLUSTALW online software (https://www.genome.jp/tools‐bin/clustalw) and ESPript 3.0 service (https://espript.ibcp.fr/ESPript/cgi‐bin/ESPript.cgi) with default parameters. The 3D structures of PAT and proteins were obtained from the Zinc database (http://zinc15.docking.org/) and the AlphaFold protein database (https://alphafold.ebi.ac.uk/), respectively. The quality of the structure was verified by SAVES v6.1 (https://saves.mbi.ucla.edu/). Semi‐flexible molecular docking was conducted by AutoDock 4.2.6. Subsequently, the docking outcomes were visualized by PyMOL 2.6.

### Plasmid Construction and Protein Expression

The potential enzyme for degrading PAT was expressed using a recombinant plasmid constructed through seamless cloning technology. First, the gene sequences encoding MgSDR1 with *Bam*HI and *Xho*I restriction sites were amplified by polymerase chain reaction (PCR) and constructed into the double‐digested vector pET‐30a (+) by ClonExpress II One Step Cloning Kit (Vazyme, Nanjing, China). Primers are listed in Table S7 (Supporting Information). Second, 10 µL ligation solution was transformed into *E. coli* DH5α. After that, the positive transformants were screened to extract the recombinant plasmid. Finally, the recombinant plasmid was transformed into *E. coli* Rosetta (DE3) to express the target protein. The expression of proteins was induced according to a modified protocol based on Zhang et al.^[^ ^21^ ^]^ Initially, *E. coli* Rosetta (DE3) containing the recombinant plasmid was cultured in 5 mL of Luria‐Bertani (LB) medium supplemented with kanamycin (50 µg mL^−1^) at 180 rpm for 12–16 h at 37 °C to prepare seed culture. The seed culture was then transferred to 100 mL of LB medium supplemented with kanamycin (50 µg mL^−1^) at a volume ratio of 1:100 and incubated at 37 °C at 180 rpm until the OD_600_ reached 0.5. Protein expression was induced by 0.5 mm IPTG, and the culture was further incubated at 120 rpm for 12 h at 16 °C. The cells were collected by centrifugation at 8000 × *g* for 20 min and subsequently washed three times with sterile water. The cells were resuspended in 10 mL of 50 mm sodium phosphate buffer (PB, pH 6.0) and disrupted by sonication. Following centrifugation at 8000 × *g* for 20 min at 4 °C, the resulting pellet and supernatant were separated, and the expression was confirmed by SDS‐PAGE (12%). The protein solution was concentrated and desalted with a 10 kDa Amicon Ultra Centrifugal Filter (Millipol, Boston, MA, USA). Finally, the Bradford Protein Assay Kit (Beyotime, Beijing, China) was used to determine the concentration of protein.

### Molecular Dynamics Simulation

Following the pretreatment of the complex through simulation, Gromacs 2022 was used for kinetic simulation. AMBER99SB‐ILDN was selected as the protein force field, and the Generalized Amber Force Field as the small molecule force field. The TIP3P model was used to add water to the system to establish a water tank with a size of 10 × 10 × 10 nm^3^ (the edge of the water box was at least 1.2 nm from the edge of the protein), and an ion automatic balance system was added. Particle‐mesh Ewald was used to deal with electrostatic interactions, and the steepest descent method was used to minimize the energy of the maximum number of steps (50 000 steps). Both the cutoff distance for the Coulomb force and the van der Waals radius were set at 1 nm. The system was balanced using both the regular system and the isothermal isobaric system. Then, the molecular dynamics simulation of 100 ns was carried out at room temperature and pressure. The non‐bond interaction cutoff value was set to 10 Å. The simulation temperature was controlled to 300 K by the Langevin thermostat, and the pressure was controlled to 1 bar by the Berendsen method. Rg, RMSD, SASA and RMSF were used for trajectory analysis and comparison.

### Effect of NADPH Concentration on PAT Degradation by MgSDR1 and Verification of the Degradation Product

The impact of NADPH concentration on the degradation reaction was investigated according to a modified protocol based on Zhang et al.^[^ ^21^ ^]^ Specifically, PAT (10 µg mL^−1^) and MgSDR1 (500 µg mL^−1^) were supplemented into the PB buffer (50 mm, pH 6) with 0, 0.01, 0.05, 0.1, and 0.5 mm NADPH. The samples were shaken at 20 °C and 180 rpm for 2 h and then centrifuged at 8000 × *g* for 5 min to pellet any cellular debris. The supernatant was then passed through filters with a pore size of 0.22 µm to remove particulates. The residual concentration of PAT was analyzed by HPLC (Agilent, Santa Clara, CA, USA) with a mobile phase of acetonitrile/water (10/90, v/v) at 1 mL min^−1^. LC‐ESI/MS (Thermofisher, Waltham, MA, USA) with a mobile phase of acetonitrile/water (10/90, v/v) for gradient elution at 40 µL s^−1^ was used to identify the PAT and its product. Scanning was performed in negative ion mode with a scan range of 50–300 m z^−1^.

### Effect of Reaction Conditions, Metal Ions, and Organic Reagents on PAT Degradation by MgSDR1

The effect of reaction conditions, organic reagents, and metal ions on the degradation reaction was investigated using a modified method derived from Zhang et al.^[^ ^21^ ^]^ Specifically, pH (3.0−6.6), temperatures (4−45 °C), the amount of enzyme (50−500 µg mL^−1^), the initial PAT concentrations (1−20 µg mL^−1^), metal ions (Fe^3+^, K^+^, Mg^2+^, Zn^2+^, Cu^2+^, Ca^2+^, Na^+^, and Mn^2+^) and organic reagents (SDS, PMSF, and EDTA) were explored. After incubation for 2 h, the samples were centrifuged at 8000 × *g* for 5 min to pellet any cellular debris. The supernatant was then passed through filters with a pore size of 0.22 µm to remove particulates. HPLC was used to detect the residual concentration of PAT. The experiment was carried out twice, with each condition being tested three times.

### PAT Degradation Assay in Fresh Pear Juices

The degradation of PAT by MgSDR1 in fresh pear juice was determined according to a modified protocol based on Xing et al.^[^ ^12^ ^]^ Fresh pear juice (pH 5.4) was made from dangshan pear. PAT (5 µg mL^−1^), NADPH (0.5 mm), and MgSDR1 (500 µg mL^−1^) were supplemented into juices and then kept at 25 °C, 180 rpm for 12 h. After incubation, the samples were centrifuged at 8000 × *g* for 5 min. The supernatant was then passed through filters with a pore size of 0.22 µm to remove particulates. HPLC was used to detect the residual concentration of PAT.

### Quality Determination of Fresh Pear Juice

Investigating how the biodegradation process impacts the quality of fruit juice is crucial. The color characteristics L* (indicating lightness), a* (spanning from red to green), b* (spanning from yellow to blue), and total color difference (Δ*E*) of the fresh pear juice were measured by a colorimeter. Cloudiness and browning index were determined following a modified method reported by Oladunjoye et al.^[^ ^30^ ^]^ pH and the contents of total phenolic, Vc, titratable acidity, and soluble solids were determined following a modified method reported by Zhang et al.^[^ ^21^ ^]^ PPO activity was measured following a modified method reported by Silva et al.^[^ ^43^ ^]^ SOD activity was measured following a modified method reported by Habibi et al.^[^ ^32^ ^]^ DPPH and ABTS free radical scavenging capacity were determined following a modified method reported by Salman et al.^[^ ^36^ ^]^ The aroma compounds were determined by GC/MS‐TQ8040 (Shimadzu, Kyoto, Japan) following a modified method reported by Shi et al.^[^ ^44^ ^]^

### Site‐Directed Mutation

The three‐step PCR technique was employed to accomplish site‐directed mutagenesis, following the method established by Andreasson et al.^[^ ^30^ ^]^ The related primers are shown in Table S8 (Supporting Information). The recombinant mutant proteins MgSDR1^S174A^, MgSDR1^Y188A^, and MgSDR1^K192A^ were expressed and purified as described in Section 2.6. The capacity of the mutant proteins to degrade PAT was assessed.

### Statistical Analysis

SPSS 23 was used for analysis of variance (ANOVA). Duncan's multiple range tests and independent samples *t*‐test were utilized to analyze statistically significant differences between groups. *p* < 0.05 indicated that the difference was statistically significant. Data were expressed as mean ± SD.

## Conflict of Interest

The authors declare no conflict of interest.

## Author Contributions

All persons have made substantial contributions to the work reported in this manuscript. Y.Z. performed conceptualization, investigation, and validation, wrote the original draft, and funded the acquisition. Q.Z. performed methodology, and data curation. S.D., E.A.G., and Y.Z. wrote, reviewed, and edited. X.B. performed methodology and data curation. Q.Y. and H.Z. performed conceptualization, supervision, and funding acquisition.

## Supporting information



Supporting Information

## Data Availability

The data that support the findings of this study are available from the corresponding author upon reasonable request.
