# Supplementary material for: Identification and Application of a Novel Patulin Degrading Enzyme From Meyerozyma guilliermondii
Source: Adv Sci (Weinh). 2025 Apr 25;12(25):2501146. doi: 10.1002/advs.202501146 (PMC12225012; doi:10.1002/advs.202501146)
Supplement: Supplementary file 1 — Supporting Information [file ADVS-12-2501146-s001.docx]

Supporting Information

Identification and application of a novel patulin degrading enzyme from *Meyerozyma guilliermondii*

Yu Zhang, Qianhua Zhao, Solairaj Dhanasekaran, Esa Abiso Godana, Yue Zhang, Xue Bai, Qiya Yang*, and Hongyin Zhang*

**Table S1.** Analysis of differentially expressed genes.

| Classification | Gene ID | Gene Name | Log_2_Fold Change (24/36 h) | | Description |
| --- | --- | --- | --- | --- | --- |
| Degrading enzymes | PGUG_04009 | *YJR096W* | 3.64/2.44 | Aldo-keto reductase | |
|  | PGUG_05193 | *SPCC663.09c* | 1.02/1.76 | Short-chain dehydrogenase/reductase family oxidoreductase | |
|  | PGUG_05170 | *ygcW* | 3.70/1.02 | Short-chain dehydrogenase/reductase family oxidoreductase | |
|  | PGUG_05194 | *SPCC663.06c* | 1.03/1.01 | Short-chain dehydrogenase/reductase family oxidoreductase | |
| Transport proteins | PGUG_03271 | *Mdr1* | 6.15/7.19 | Major facilitator superfamily transporter | |
|  | PGUG_05048 | *Mdr1* | 2.92/1.79 | Major facilitator superfamily transporter | |
|  | PGUG_04884 | *Azr1* | 1.31/3.04 | Multi-drug resistance family major facilitator superfamily transporter | |
|  | PGUG_01265 | *Azr1* | 1.46/1.98 | Multi-drug resistance family major facilitator superfamily transporter | |
|  | PGUG_03615 | *Opt2* | 1.19/1.21 | OPT family oligopeptide transporter | |
| Zinc finger transcription factors | PGUG_03272 | *Mrr1* | 1.20/1.36 | Zn(II)_2_Cys_6_ transcription factor | |
|  | PGUG_05155 | *Stb4* | 1.21/1.32 | Zn(II)_2_Cys_6_ transcription factor | |
|  | PGUG_05406 | *Crz2* | 1.43/1.57 | C_2_H_2_-type zinc finger | |
| Oxidative stress  Glutathione  metabolism | PGUG_03124 | *Gcs1* | 1.07/1.05 | Glutamate-cysteine ligase | |
|  | PGUG_01005 | Gsh2 | 1.18/1.76 | Glutathione synthetase | |
|  | PGUG_03124 | Gcs1 | 1.07/1.05 | Glutamate-cysteine ligase | |
|  | PGUG_01005 | Gsh2 | 1.18/1.41 | Glutathione synthetase | |

**Table S1.** Analysis of differentially expressed genes (continued).

| Classification | Gene ID | Gene Name | Log_2_Fold Change (24/36 h) | | Description |
| --- | --- | --- | --- | --- | --- |
|  | PGUG_02918 | *Rnr1* | 1.31/2.09 | Ribonucleoside-diphosphate reductase large subunit (subunit alpha) | |
|  | PGUG_02981 | *Rnr2* | 1.01/1.42 | Ferritin family protein | |
| Growth and reproduce | PGUG_01988 | *Asf1* | 1.01/2.13 | Anti-silencing function 1 family histone chaperone | |
|  | PGUG_02099 | *Epd1* | 1.01/1.70 | Glyco_hydro_72 and X8 domain-containing protein | |
| DNA replication | PGUG_00348 | *Mcm2* | 1.10/1.97 | DNA replication licensing factor miniature chromosome maintenance | |
|  | PGUG_02112 | *Mcm7* | 1.26/2.30 | DNA replication licensing factor miniature chromosome maintenance | |
|  | PGUG_02276 | *Mcm5* | 1.19/2.12 | DNA replication licensing factor miniature chromosome maintenance | |
|  | PGUG_03230 | *Mcm4* | 1.34/3.88 | DNA replication licensing factor miniature chromosome maintenance | |
|  | PGUG_04124 | *Pri2* | 1.15/2.14 | DNA primase large subunit | |
|  | PGUG_01613 | *Pol2* | 1.49/2.35 | DNA polymerase epsilon catalytic subunit A | |
|  | PGUG_00042 | *Sld2* | 1.15/1.47 | Drc1-Sld2 domain-containing protein | |
| DNA damage repair | PGUG_00290 | *Psm1* | 1.58/3.23 | Structural maintenance of chromosomes superfamily proteins | |
|  | PGUG_04903 | *Dot1* | 1.05/1.04 | S-adenosylmethionine-dependent methyltransferases | |
|  | PGUG_05709 | *Ctf18* | 1.12/2.05 | ATP-binding protein | |
|  | PGUG_05723 | *Rad21* | 1.27/7.24 | Rad21/Rec8 family protein | |

**Table S2.** Physical and chemical properties of potential degrading enzymes.

| Proteins | Number of amino acids | | Molecular weight/kDa | | Theoretical  pI | | Molecular formula | Instability  index | | Grand average of  hydropathicity | | Aliphatic index | |
| --- | --- | --- | --- | --- | --- | --- | --- | --- | --- | --- | --- | --- | --- |
| MgAKR | | 287 | | 31.09 | | 8.98 | C_1484_H_2333_N_399_O_430_S_10_ | | 41.74 | | -0.474 | | 85.54 |
| MgSDR1 | | 290 | | 32.96 | | 5.97 | C_1360_H_2190_N_378_O_431_S_11_ | | 38.59 | | 0.024 | | 95.45 |
| MgSDR2 | | 250 | | 27.29 | | 6.12 | C_1222_H_1927_N_323_O_374_S_5_ | | 14.52 | | -0.158 | | 88.24 |
| MgSDR3 | | 251 | | 27.12 | | 5.61 | C_1223_H_1957_N_317_O_370_S_3_ | | 22.12 | | 0.052 | | 104.86 |

**Table S3.** Effect of biodegradation process on color parameters (*L**, *a**, *b** and Δ*E*), cloudiness and non-enzymatic browning index on fresh pear juice.

| Treatment | *L** | *a** | *b** | Δ*E** | Cloudiness | Browning index |
| --- | --- | --- | --- | --- | --- | --- |
| Before degradation | 33.73±2.05^a^ | 0.19±0.00^a^ | -0.18±0.48^a^ | 61.10±2.02^a^ | 0.20±0.00^a^ | 0.12±0.00^a^ |
| After degradation | 32.80±2.16^a^ | 0.13±0.05^a^ | -0.28±0.26^a^ | 62.02±2.13^a^ | 0.20±0.00^a^ | 0.12±0.00^a^ |

^a)^Means with different letters show significantly different at *P* < 0.05 according to the independent sample t-test.

**Table S4.** GC/MS analytical results of aromatic compounds in fresh pear juice before and after enzymatic reaction.

| Number | Name | Peak time (min) | Percentage (%) | |
| --- | --- | --- | --- | --- |
|  |  |  | Before degradation | After degradation |
| 1 | Hexanal | 5.56 | 5.92 | 8.66 |
| 2 | 1-Hexanol | 7.89 | 1.15 | 1.7 |
| 3 | 3-Hexen-1-ol, acetate, (*Z*)- | 11.84 | 1.4 | 1.83 |
| 4 | Acetic acid, hexyl ester | 12.03 | 1.00 | 1.47 |
| 5 | Nonanal | 14.34 | 1.41 | 1.35 |
| 6 | Decanal | 16.65 | 0.43 | 0.52 |
| 7 | 1-Dodecanol | 21.94 | 0.12 | 0.07 |

**Table S5**. Kinetic parameters of MgSDR1.

| Catalyst | MgSDR1 |
| --- | --- |
| *K*m (mM) | 1.42 |
| *V*max (×10^-2^, μM s^-1^) | 19.84 |
| *K*cat (×10^-3^, s^-1^) | 12.3 |
| *K*cat/*K*m (s^-1^ M^-1^) | 8.66 |

**Table S6.** Primers used for RT-qPCR.

| Primers | Sequences (5’-3’) |
| --- | --- |
| PGUG_05193-F | TTCTGATTGCGGCTCGTTAAACTTG |
| PGUG_05193-R  PGUG_05194-F  PGUG_05194-R | GCTCCAACTGTCTCGCCAACTC  GCGGTTCATCCTGGTGTAGTTGG  CACTCAGCGAGAAGAAGTCAGCAG |
| PGUG_05170-F | AGTCACCAGTGTCGTCGGAGATC |
| PGUG_05170-R | CTCACCTGGCGTGTAACTGATAGC |
| PGUG_04009-F | ATTGCCAATTCCAAGGAGGAGGTG |
| PGUG_04009-R | ACTACGCTGTCGATGTCCCAAATTC |
| PGUG_01005-F | TTCGGAACTTGCTCGTGGTCTTG |
| PGUG_01005-R | CGCTCGCCAGGTTGAACAATTAC |
| PGUG_03124-F | ATAAGGTGGATGCCGTGTTGAAGG |
| PGUG_03124-R | CGTCGTTGCCGTGGTTGATAAAC |
| PGUG_02918-F | CGGTGCTGTTGAGGCTTCTATCG |
| PGUG_02918-R | ATCCCAGTCCCACAACTCAGTAGG |
| PGUG_02981-F | GCTTGCTTGTTGTTCTCCCACTTG |
| PGUG_02981-R | TCCTTCTCAATGTCAACGGCTTCG |
| β-tublin-F | CCAATTCCTGGCTCCGCTTACG |
| β-tublin-R | CTGTCCTACTTCCGCACCCTTTTC |

| Primers | Sequences (5’-3’) |
| --- | --- |
| pET-30a-*MgYJR096W*-F | gccatggctgatatcggatccATGACTTCCCTCACCATGAA |
| pET-30a-*MgYJR096W*-R  pET-30a-*MgygcW*-F  pET-30a-*MgygcW*-R  pET-30a-*MgSPCC663.09c*-F  pET-30a-*MgSPCC663.09c*-R  pET-30a-*MgSPCC663.06c*-F  pET-30a-*MgSPCC663.06c*-R | gtggtggtggtggtgctcgagGTCCTGGTACGTCGTGGG  gccatggctgatatcggatccATGTCATTAAGGGTTTCA  gtggtggtggtggtgctcgagATCATCTTGAGATATAT  gccatggctgatatcggatccATGGAACAAACGTACTTT  gtggtggtggtggtgctcgagCCATGGAAGTTCGGTTCC  gccatggctgatatcggatccATGACAAAGACTTATTTT  gtggtggtggtggtgctcgagCTACCATGGGAGTTCGGA |

**Table S****7.** Primers used for degrading enzymes encoding gene cloning.

^a)^Small letters denote the homologous recombination sequences of the vector pET-30a (+).

**Table S8.** Primers used for site-directed mutagenesis of MgSDR1.

| Primers | Sequences (5’-3’) |
| --- | --- |
| S174A-F  S174A-R  Y188A-F  Y188A-R  K192A-F  K192A-R | AATATGCTTT**GCT**TCTGTGTCCT  AGGACACAGA**AGC**AAAGCATATT  TGTTCCGTCT**GCT**CAAACCTCA  TGAGGTTTG**AGC**AGACGGAACA  CAAACCTCA**GCA**GCCTCTGTTG  CAACAGAGGC**TGC**TGAGGTTTG |

^a)^Bold letters denote the mutation sites.

**

**

**Figure S1.** Validation of predicted structure of protein using several evaluation methods such as ERRAT and PROCHECK. A. MgSDR1. B. MgSDR2. C. MgSDR3. D. MgAKR. E. MgGCS1. F. MgGSH2. G. MgRNR1. E. MgRNR2.

**
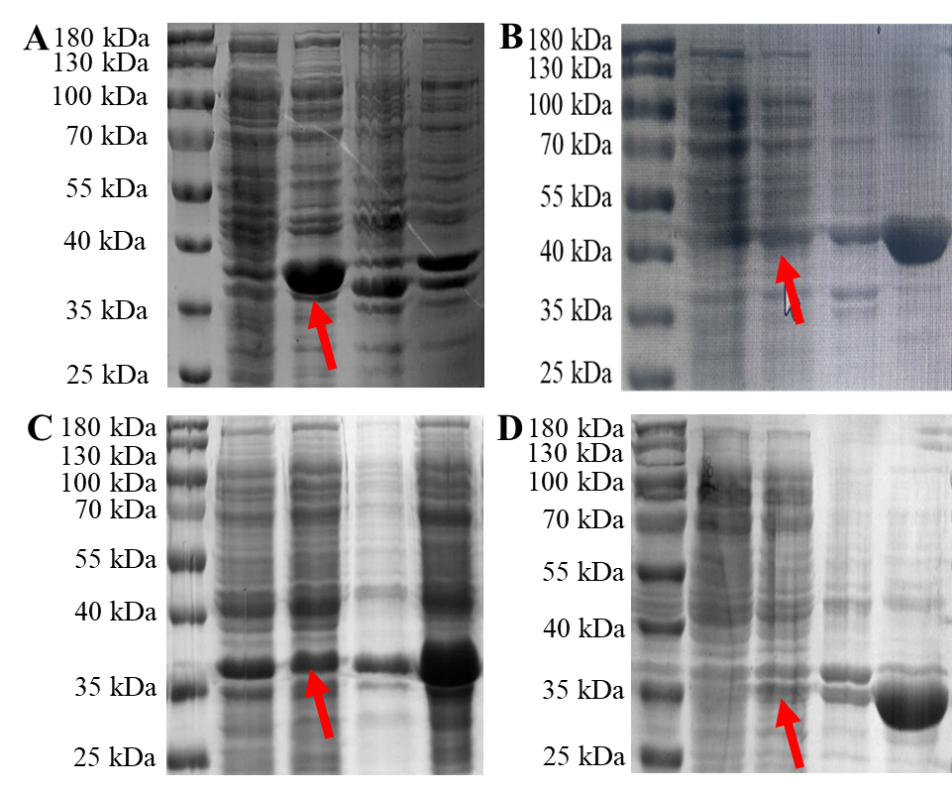
**

**Figure S2.** Expression and purification of potential degrading enzymes (A) YJR096W encoding MgAKR. (B) ygcW encoding MgSDR1. (C) SPCC663.09c encoding MgSDR2. (C) SPCC663.06c encoding MgSDR3. Lane 1: PageRuler prestained molecular weight marker, Lanes 2 and 4: soluble proteins and insoluble inclusion bodies from the *E. coli* strain without IPTG induction, Lanes 3 and 5: soluble proteins and insoluble inclusion bodies from the *E. coli* strain with IPTG induction.

**
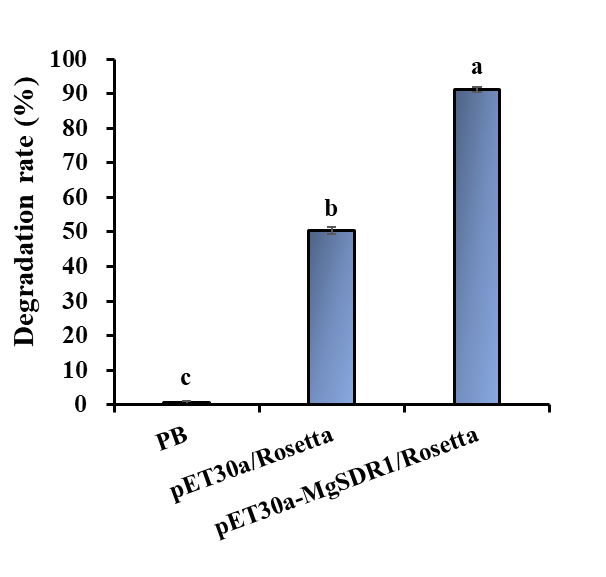
**

**Figure S3** Ability of the MgSDR1-expressed *E. coli* Rosetta (DE3) strain to degrade PAT. The data are the means ± standard deviations. Values followed by different letters are significantly different according to Duncan’s multiple range test (*P* < 0.05).
